# Transcriptome analysis in calorie-restricted rats implicates epigenetic and post-translational mechanisms in neuroprotection and aging

**DOI:** 10.1186/s13059-015-0847-2

**Published:** 2015-12-22

**Authors:** Shona H. Wood, Sipko van Dam, Thomas Craig, Robi Tacutu, Amy O’Toole, Brian J. Merry, João Pedro de Magalhães

**Affiliations:** Integrative Genomics of Ageing Group, Institute of Integrative Biology, University of Liverpool, Liverpool, UK

**Keywords:** Epigenetics, lifespan, longevity, nutrigenomics, RNA-seq

## Abstract

**Background:**

Caloric restriction (CR) can increase longevity in rodents and improve memory function in humans. α-Lipoic acid (LA) has been shown to improve memory function in rats, but not longevity. While studies have looked at survival in rodents after switching from one diet to another, the underlying mechanisms of the beneficial effects of CR and LA supplementation are unknown. Here, we use RNA-seq in cerebral cortex from rats subjected to CR and LA-supplemented rats to understand how changes in diet can affect aging, neurodegeneration and longevity.

**Results:**

Gene expression changes during aging in ad libitum-fed rats are largely prevented by CR, and neuroprotective genes are overexpressed in response to both CR and LA diets with a strong overlap of differentially expressed genes between the two diets. Moreover, a number of genes are differentially expressed specifically in rat cohorts exhibiting diet-induced life extension. Finally, we observe that LA supplementation inhibits histone deacetylase (HDAC) protein activity *in vitro* in rat astrocytes. We find a single microRNA, miR-98-3p, that is overexpressed during CR feeding and LA dietary supplementation; this microRNA alters HDAC and histone acetyltransferase (HAT) activity, which suggests a role for HAT/HDAC homeostasis in neuroprotection.

**Conclusions:**

This study presents extensive data on the effects of diet and aging on the cerebral cortex transcriptome, and also emphasises the importance of epigenetics and post-translational modifications in longevity and neuroprotection.

**Electronic supplementary material:**

The online version of this article (doi:10.1186/s13059-015-0847-2) contains supplementary material, which is available to authorized users.

## Background

Ageing is arguably the major biomedical challenge of the 21^st^ century with the incidence of age-related diseases, and neurodegenerative diseases in particular, expected to increase dramatically in the coming decades. Brain ageing frequently underlies cognitive decline and is a major risk factor for neurodegenerative conditions such as Alzheimer's disease (AD) and Parkinson's disease. Mental health is also a major concern of ageing adults. The exact molecular mechanisms underlying brain ageing, however, remain unknown [1].

A major tool available to researchers studying ageing is caloric restriction (CR), which consists of restricting food intake (without malnutrition) of organisms normally fed ad libitum (AL). CR is the only dietary intervention known to increase longevity and retard the process of ageing in several model organisms. In mice and rats, CR can increase longevity by ~30 %, delay physiological ageing and postpone or diminish the morbidity of most age-related diseases [2]. CR can also delay brain iron accumulation, preserving motor performance, and in one study increased survival in rhesus monkeys [3, 4]. However, as reported for rodent species [5, 6], there is significant variation in the mortality effects of CR feeding in different studies using rhesus monkeys [7, 8]. In elderly humans, CR has been shown to improve memory function [9].

α-Lipoic acid (LA) is an anti-oxidant which naturally occurs in many foods. It does not increase survival in rodent studies [10], but rats supplemented with LA during the AL phase of feeding when switched from CR to AL feeding at 12 months of age retained the extended longevity characteristic of CR feeding throughout life despite a return to AL feeding. This was termed a dietary memory effect [10]. These animals gained weight, indicating that although the longevity effects of CR were preserved, other effects of CR feeding were not. Conversely, animals switching from AL, with a diet supplemented with LA, to CR feeding without LA supplementation did not exhibit extended longevity, yet extended survival is a normal response of this feeding switch when LA is not included in the diet during AL feeding. This LA-induced memory effect gives us a unique opportunity to identify the mechanisms by which CR retards ageing and increases longevity without the confounding physiological effects of CR (e.g. restricted growth). Furthermore, LA can compensate for age-related, long-term memory deficits in old rats [11] and improve memory and learning in prematurely aged mice [12].

The underlying mechanisms of the beneficial effects of CR and LA supplementation are unknown. Understanding how these changes in diet and feeding regime can affect ageing, neurodegeneration and longevity is of great importance and could pinpoint key genes and pathways for further research relevant to human application [13]. The aim of this work was to study the transcriptomes, using RNA-seq, of the cerebral cortex of rats subjected to CR and LA-supplemented rats and compare them with animals maintained on an AL diet not supplemented with LA.

Using rat cerebral cortex from a previous study [10], which identified the dietary memory effect, our results show changes in expression of genes related to secretory and neuroendocrine pathways with ageing in AL-fed rats, many of which are prevented by CR. We find also that CR and LA supplementation share a largely common gene expression profile, despite the different effects on longevity. These shared genes are mainly neuroprotective and may act through prevention of excitoxicity, by down-regulation of glutamate receptors and calcium kinases, and inducing protection against oxidative stress by the up-regulation of glutathione S-transferases (GSTs) and thioredoxin. We also propose that this neuroprotective effect of CR and LA is driven by changes in histone deacetylase (HDAC) and histone acetyltransferase (HAT) homeostasis and implicate a miRNA (miR-98-3p) in this process. This was demonstrated using transfection, cell culture and HDAC/HAT activity assays. We show that genes involved in acetylation of histones are altered and may be important in the dietary memory effect of LA on longevity. Finally, we identify 137 genes that are associated with rat cohorts exhibiting diet-induced longevity. Many of these are evolutionarily conserved in mouse, worm and fly longevity networks.

## Results

Using tissues from a previous study [10], we performed RNA-seq on the cerebral cortex of rats. Three basic feeding/dietary groups were established: rats fed AL throughout life; rats subjected to CR throughout life; or rats fed a LA-supplemented diet during the AL feeding phase before switching to or from CR feeding at 12 months of age. The longevity of each of these groups of animals and the number of differentially expressed (DE) genes are shown in Table 1. To establish the effect of ageing on the transcriptome and the impact of CR on age-related changes, rats were sampled at 6, 12 and 28 months of age.

**Table 1:** Mean survival of rats on the various diets used in this study and the number of DE genes compared with AL feeding

Rat cohorts^a^	Mean survival (days) from [10]	SE (days)	Number of DE genes compared with AL
AL	854	22	NA
*AL > CR	1000	33	1028
AL + LA > CR	859	57	986
*CR	1025	25	941
CR > AL	914	44	1435
*CR > AL + LA	1009	34	1233
AL + LA	858	27	1003

^a^Diet switches are indicated by “>” and occurred at 12 months of age. Asterisks (*) indicate long-lived cohorts, all statistically significant versus AL. Benjamini-Hochberg false discovery rate <0.05; n = 6 per group (pooled two by two) for the RNA-seq; 10,620 genes expressed in total. *NA* not applicable, *SE* standard error

### Changes observed in the ageing transcriptome are prevented by a calorie restricted diet in rats

We have previously published an analysis of whole transcriptome RNA-seq for an AL-fed ageing cohort (6, 12 and 28 months) [14]. In the current study we re-sequenced the AL-fed ageing cohort at a greater depth on the 5500XL SOLiD system (previously we used the SOLiD system v4), along with all the other experimental groups in this study to eliminate any platform bias. The major findings from the previous analysis were confirmed and the number of DE genes was similar, showing good replication (data not shown), but due to the updated annotation of the rat genome we found also a number of new DE genes.

**Fig. 1:**
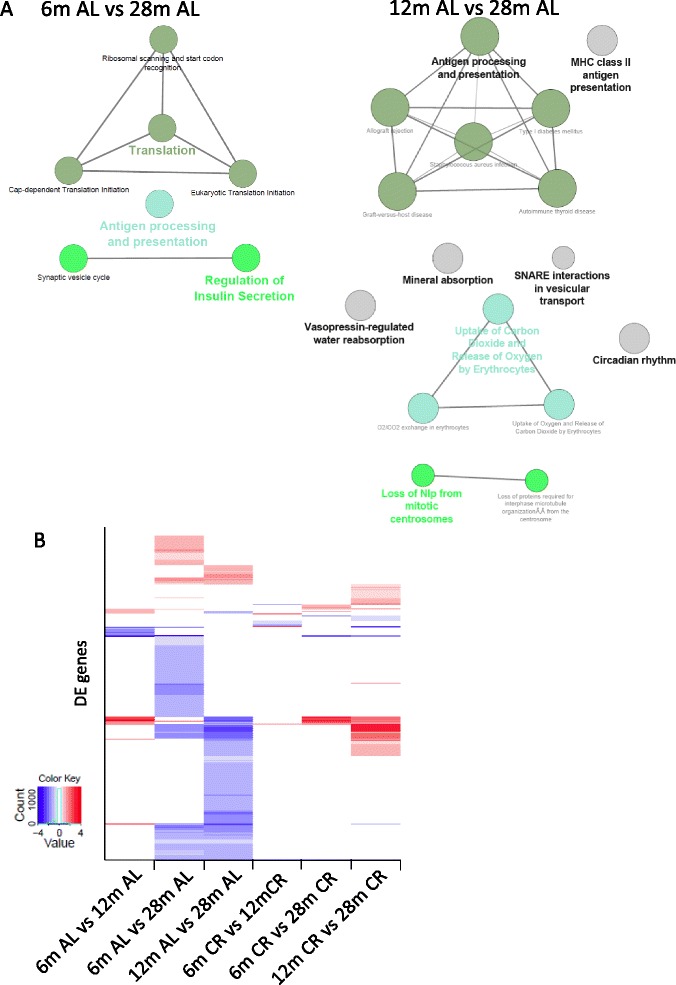
Age-related differential gene expression in rats fed AL and subjected to CR. **a** Two simplified networks of related, statistically significant enriched Gene Ontology (GO) terms using the Cytoscape add-on ClueGO [30, 31]. The network comparing 6 months AL (*6 m AL*) and 28 months AL *(28 m AL*) is shown on the left and 12 months AL (*12 m AL*) and 28 months AL (*28 m AL*) is shown on the right. DE genes were used to generate a GO term network. The *filled coloured circles* (nodes) represent each statistically significant parent GO term. The *lines* (edges) between the nodes show that there are overlapping genes between terms. The Cytoscape add-on ClueGO allows enrichment analysis and the collapsing of GO terms into parent categories for each comparison. Each of the terms is statistically significant (Benjamini-Hochberg correction <0.05). Colours represent shared GO terms. The different sizes of the nodes relate to how many genes fall into the terms. **b** Heatmap of the DE genes with age across all AL and CR datasets (Table 2); created in R using the heatmap3 package. The y-axis represents all the DE genes. *Red* = up-regulated, *blue* = down-regulated, log2 fold change reported for statistically significant DE genes. *White* means no statistically significant change in expression. *6 m* = 6 months of age, *12 m* = 12 months of age, *28 m* = 28 months of age

Our new analysis emphasises an enrichment amongst DE genes with age for antigen presentation via MHC II (Fig. 1a; Additional file 1) and the down-regulation of heat shock proteins, as previously described [14]. However, statistically significant enrichment for synaptic vesicle cycle and vesicular transport related genes are also observed (Fig. 1a). Chromogranin B, which is important in secretion and neuroendocrine pathways, is down-regulated with age (6 versus 28 months and 12 versus 28 months), which is interesting as this gene is also down-regulated in AD [15]. Conversely, long-lived Snell’s dwarf mice show higher levels of chromogranin B [16]. Pcsk1n, an inhibitor of Pcsk1, which regulates the proteolytic cleavage of neuroendocrine peptide precursors, including chromogranin B [17], is under-expressed with age (12 versus 28 months). Moreover, down-regulation of genes related to synaptic transmission and synaptic vesicle transport (*syntaxin-1b*, *syntaxin-binding protein 1*, *syntaxin-1A* and *Napb*), post-synaptic signalling modulators (e.g. *Syngap1*, *Shank1*, *Homer2* and *Shank3*) and a glutamate receptor (*Grik4*) occurs with age (6 versus 28 months) in the AL cohort. These distinct changes in secretory pathways, synaptic vesicle transport and synaptic transmission are consistent with observations of neurodegenerative diseases [18–20].

Rev-erb-α (nuclear receptor 1D1) is a transcriptional repressor involved in circadian rhythmicity (an enriched term; Fig. 1a) and metabolism, which is down-regulated with age. Its mode of action is through the recruitment of the co-repressor NCoR, which is also down-regulated in our data, and activation of Hdac3 [21]. Nur77 (nuclear receptor 4A1), a transcription factor that is down-regulated, interacts with HAT, p300 and Hdac1, which regulates expression through acetylation [22]. These results are interesting because nuclear receptors are important in hormone homeostasis, sensing hormones and regulating the downstream expression of multiple genes through epigenetic mechanisms. All genes DE with age are presented in Additional file 2.

The majority of the gene expression changes observed with age in AL-fed rats are not present during ageing of CR rats (Fig. 1b), with the exception of *Hspa1b* (heat shock protein, −1.8 fold change (FC) in CR and −3.6 FC in AL groups), supporting the hypothesis that the ageing process is retarded by CR. Fewer genes are DE with age in CR than in AL (Table 2) and there are a limited number of genes which are only DE during ageing in rats subjected to CR (Fig. 1b).

**Table 2:** Comparison of the number of genes differentially expressed with age in AL and CR groups

Comparison	AL	CR
6 versus 12 months	24 (15 protein coding)	10 (10 protein coding)
6 versus 28 months	180 (162 protein coding)	17 (17 protein coding)
12 versus 28 months	174 (141 protein coding)	70 (45 protein coding)

Benjamini-Hochberg false discovery rate <0.05; n = 6 per group (pooled two by two); 10,620 genes expressed in total

Over-representation analysis (gene set enrichment analysis (GSEA), Molecular Signatures Database, Broad Institute) independently revealed that our data statistically significantly overlapped with a previous meta-analysis of ageing based on microarray studies (false discovery rate (FDR) q value = 2.12 × 10^-7^) [23] and with a list of genes DE after methyl binding protein knockdown (MeCP2, MB1 and MBD2) in Hela cells (1.8 × 10^-4^) [24]. This leads to the question: what is driving this altered gene expression profile with age?

The histone H3 methyltransferase Dot1L was downregulated with age in the AL cohort (−1.2 log FC in 6 months AL versus 28 months AL; −1.4 FC in 12 months AL versus 28 months AL). Previously, H3K79 methylation (regulated by DOT1L) was identified as a novel histone marker for ageing and cancer [25]. Tet3, an important regulator of DNA demethylation and H3K4 trimethylation [26–28], is down-regulated with age in our data (−1.2 FC in 12 months AL versus 28 months AL). Furthermore, the chromatin remodeller Chd5 (Chromodomain helicase DNA binding protein 5) was DE with age in our results and has previously been linked to ageing and AD [29].

Taken together, these results indicate that there are changes in the responsiveness of cells to hormonal signalling with age, as well as expression changes in genes related to chromatin remodelling and modification, possibly affecting transcription at various levels.

### Neuroprotective genes are overexpressed in rats in response to CR and LA-supplemented diets

**Fig. 2:**
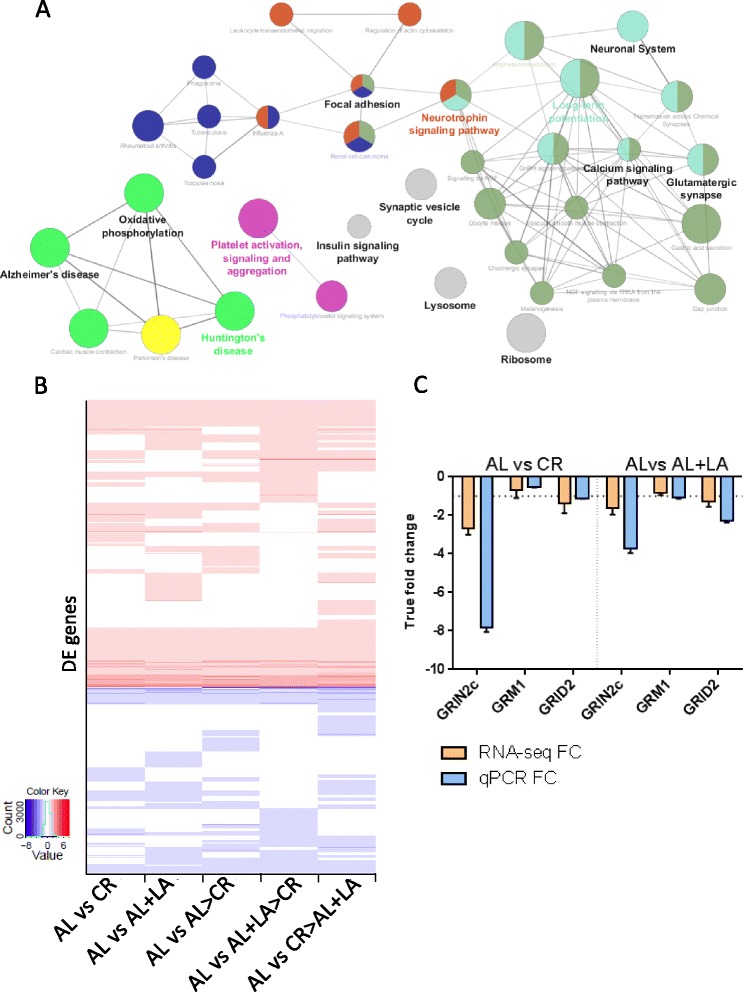
CR and LA supplementation elicit a similar neuroprotective gene expression profile. **a** Simplified networks of related statistically significant enriched GO terms using the Cytoscape add-on ClueGO [30, 31]. The network was made from the overlapping differentially expressed genes between all diets with CR and LA supplementation (a.k.a neuroprotective profile; see text for details). The Cytoscape add-on ClueGO allows enrichment analysis and the collapsing of GO terms into parent categories for each comparison. Each of the terms is statistically significant (Benjamini-Hochberg correction <0.05). The *filled coloured circles* (nodes) represent each statistically significant enriched parent GO term. The *lines* (edges) between the nodes show that there are overlapping genes within terms. The different sizes of the nodes relate to how many genes fall into the terms. **b** Heatmap of the differentially expressed genes (Table 1) across all diets with CR and LA supplementation when compared with AL; created in R using the heatmap3 package. *Red* = up-regulated, *blue* = down-regulated, log2 FC reported for statistically significant DE genes. *White* means no statistically significant change in expression. **c** Quantitative PCR confirmation of RNA-seq results, showing glutamate receptor differential expression (*GRIN2c*, *GRM1* and *GRID2*) when comparing CR (*left*) and AL supplemented with LA (*right*) with AL alone. *Orange bars* are RNA-seq FC and *blue bars* are qPCR FC. The *horizontal dotted line* represents the one FC cutoff. The *vertical dotted line* separates the CR and LA supplemented comparisons. *Error bars* represent the standard error calculated for log2 FC as follows: (Standard error/Mean) × log2e. *Hprt1*, *B2m* and *Ywhaz* were used as reference genes. N = 6 per group, unpooled RNA from the RNA-seq experiment

In order to understand the previously observed neuroprotective effects of CR and LA [3, 4, 9, 11, 12, 30], we focused on the response of rats to CR and LA-supplemented diets at 28 months of age. By directly comparing 28 months AL to 28 months CR, we found 895 DE genes enriched for glutamatergic synapse, oxidative phosphorylation and terpenoid backbone biosynthesis (Additional file 3). Furthermore, there is a 71 % overlap between genes DE in CR rats and in LA-fed rats at 28 months when compared with AL rats; this overlap is reduced to 49 % when including all the dietary variations in our study (Fig. 2b; Table 3; Additional file 4). By taking the overlapping DE genes induced by CR feeding and LA diets we generated an enrichment map based on Gene Ontology (GO) terms. Neurotrophin signalling pathway, essential in the survival of neurons through protection against excitotoxicity [31] and reported to change in the aged brain [32], is a key enriched term (Fig. 2a). Furthermore, neurotrophins protect neurons against excitotoxicity and rescue oxidative stress-mediated apoptosis by involvement of the phosphatidylinositol and mitogen-activated protein kinase (MAPK) pathways [33]. Using DAVID functional enrichment analysis we found that the majority of the down-regulated genes overlapping between CR feeding and LA diets are involved in neuroprotection (Additional file 4). Glutamate receptors and receptor activity genes were altered or enriched in our datasets, and we conducted quantitative PCR (qPCR) to confirm these findings (Fig. 2c).

**Table 3:** Shared chromatin related and epigenetic regulator genes altered by CR and LA feeding

Ensembl ID	Associated gene name	Description	AL versus CR log2 FC	AL versus CR FDR	AL versus AL+ LA log2 FC	AL versus AL+ LA FDR
ENSRNOG00000001442	Por	NADPH--cytochrome P450 reductase [Source:UniProtKB/Swiss-Prot;Acc:P00388]	1.24	8.58 × 10^−06^	0.75	1.19 × 10^−02^
ENSRNOG00000043098	Mt2A	Metallothionein-2 [Source:UniProtKB/Swiss-Prot;Acc:P04355]	1.06	7.02 × 10^−04^	1.30	4.81 × 10^−05^
ENSRNOG00000048961	Bhlhe41	Bhlhe41	1.06	5.45 × 10^−04^	0.92	7.17 × 10^−03^
ENSRNOG00000047374	Gnas	GNAS isoform GNASL [Source:RefSeq peptide;Acc:NP_062005]	0.99	2.69 × 10^−08^	0.90	1.73 × 10^−05^
ENSRNOG00000008001	Rab3b	Ras-related protein Rab-3B [Source:UniProtKB/Swiss-Prot;Acc:Q63941]	0.89	4.16 × 10^−04^	0.57	3.70 × 10^−02^
ENSRNOG00000018958	Mt3	Metallothionein-3 [Source:UniProtKB/Swiss-Prot;Acc:P37361]	0.77	3.94 × 10^−03^	0.58	3.88 × 10^−02^
ENSRNOG00000005183	Rtf1	RNA polymerase-associated protein RTF1 homolog [Source:RefSeq peptide;Acc:NP_001102428]	0.58	4.50 × 10^−03^	0.73	4.32 × 10^−05^
ENSRNOG00000006816	Urb5	Ubiquitin protein ligase E3 component n-recognin 5 [Source:MGI Symbol;Acc:MGI:1918040]	−0.43	3.64 × 10^−02^	−0.44	2.97 × 10^−02^
ENSRNOG00000013837	Trp53bp1	Transformation related protein 53 binding protein 1 [Source:MGI Symbol;Acc:MGI:1351320]	−0.50	1.48 × 10^−02^	−0.53	9.34 × 10^−03^
ENSRNOG00000005544	Kansl1	KAT8 regulatory NSL complex subunit 1 [Source:MGI Symbol;Acc:MGI:1923969]	−0.53	2.46 × 10^−02^	−0.61	1.26 × 10^−02^
ENSRNOG00000004289	Kdm6a	Lysine (K)-specific demethylase 6A [Source:MGI Symbol;Acc:MGI:1095419]	−0.59	1.24 × 10^−02^	−0.55	7.33 × 10^−03^
ENSRNOG00000003220	H3f3c	Histone H3.3 [Source:UniProtKB/Swiss-Prot;Acc:P84245]	−0.72	4.50 × 10^−02^	−0.75	1.50 × 10^−02^
ENSRNOG00000011523	H2afy	Core histone macro-H2A.1 [Source:UniProtKB/Swiss-Prot;Acc:Q02874]	−0.73	2.56 × 10^−03^	−0.82	5.60 × 10^−04^
ENSRNOG00000015781	Ndst3	Bifunctional heparan sulfate N-deacetylase/N-sulfotransferase 3 [Source:RefSeq peptide;Acc:NP_001178645]	−0.82	4.27 × 10^−03^	−0.77	8.10 × 10^−03^
ENSRNOG00000046636	HIST1H4B	Histone H4 Osteogenic growth peptide	−0.92	6.85 × 10^−04^	-1.27	8.54 × 10^−08^
ENSRNOG00000017714	Usp3	Ubiquitin carboxyl-terminal hydrolase 3 [Source:RefSeq peptide;Acc:NP_001020595]	−0.99	1.11 × 10^−02^	−0.99	1.37 × 10^−02^
ENSRNOG00000006689	Chd7	Chromodomain helicase DNA binding protein 7 [Source:MGI Symbol;Acc:MGI:2444748]	-1.09	3.92 × 10^−03^	−0.93	1.60 × 10^−02^
ENSRNOG00000014397	Zic2	zinc finger protein ZIC 2 [Source:RefSeq peptide;Acc:NP_001101862]	-1.81	5.00 × 10^−04^	-2.06	7.44 × 10^−05^

Benjamini-Hochberg FDR <0.05; n = 6 per group (pooled two by two)

Strong enrichment of oxidative phosphorylation (DAVID enrichment score (ES) = 5.44, FDR = 8.93 × 10^-10^) and AD was found in the 490 shared up-regulated genes in CR and LA diets (Fig. 2a; Additional file 4). GSTs are a family of enzymes that play an important role in detoxification and protect neurons from oxidative insult [34]. During CR feeding and LA supplemented diets, four GSTs were up-regulated (*Gsta1*, *Gstm1*, *Gstp1*, *Gstt3*). Furthermore, thioredoxins are redox proteins that respond to reactive oxygen species, therefore acting as antioxidants, and three are up-regulated in response to CR feeding and LA supplementation (*Txndc17*, *Txnip* and *Txnrd1*). Oxidative stress is thought to be important in ageing, in particular in energy-rich tissues such as the brain, promoting degenerative disease and accelerating ageing [35]; therefore, up-regulation of antioxidant enzymes may be neuroprotective. Overall, changes in synaptic transmission and susceptibility to glutamate excitotoxicity have been described as a potential cause of neurodegeneration [36]. Our results suggest that CR and LA feeding induce gene expression changes that prevent excitotoxicity through down-regulation of glutamate receptors and calcium kinases, and protect against oxidative stress, inducing a neuroprotective profile.

We set out to identify the genes regulating this neuroprotective profile and noticed that a large number of chromatin-related and epigenetic regulators were altered due to CR feeding and LA supplementation (Table 3). This neuroprotective profile may be driven through changes in epigenetic regulators altering the acetylation and methylation status of DNA, histones and proteins. However, there are a number of epigenetic regulators that are DE specifically in response to either CR feeding or LA supplementation, and we predicted that there would be a shared regulator of this neuroprotective profile.

**Fig. 3:**
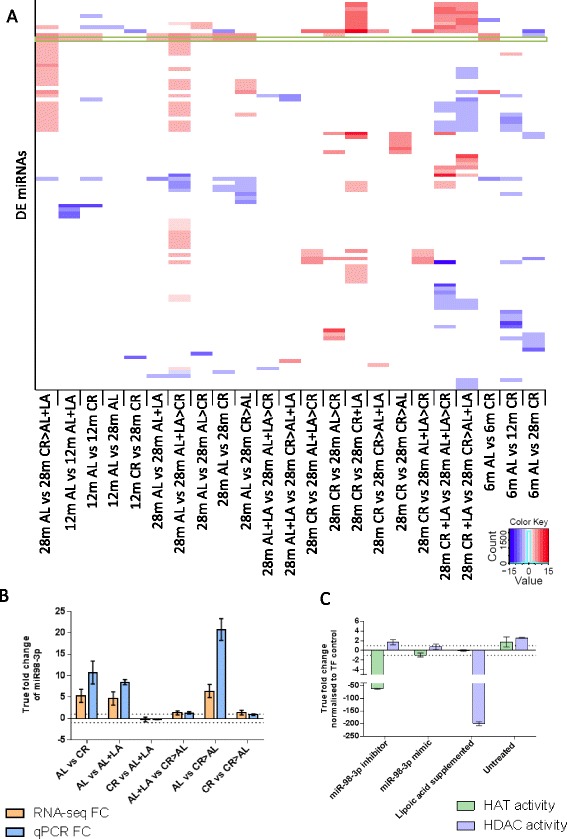
Each diet group and age elicits its own miRNA expression profile but miR-98-3p is overexpressed in all CR and LA diets and may affect HAT and HDAC activity. **a** Heatmap of the 175 DE miRNAs across all diets and ages; created in R using the heatmap3 package. mir-98-3p is highlighted by a *green box*. Red = up-regulated, and blue = down-regulated, log2 fold change reported for statistically significant DE genes. White means no statistically significant change in expression. **b** qPCR confirmation of RNA-seq results, showing miR98-3p differential expression in all CR and LA supplemented diets. *Orange bars* are RNA-seq log2 FC and *blue bars* are qPCR FC (corrected to provide the true FC in the sample by Ratio – 1 if the sample was up-regulated and by (–1/Ratio) + 1 for down-regulated samples). The *horizontal dotted line* represents the 1 FC cutoff. *Errors bars* represent the standard error calculated for log2 FC as follows: (Standard error/Mean) × log2e. *Snord96a*, *Snord95* and *Snord68* were used as reference genes. **c** The difference in HDAC and HAT activity was calculated as a ratio of the transfection (*TF*) control and then transformed into true FC (by Ratio – 1 if the sample was up-regulated and by (–1/Ratio) + 1 for down-regulated samples). A cutoff of 1 FC was used, represented by the *dotted line*. The figure shows that the miR-98-3p inhibitor significantly (*p* < 0.001, two-tailed t-test) decreases HAT activity when compared with HDAC activity. A miR-98-3p mimic appears not to affect HDAC/HAT balance (no statistically significant difference). LA reduces HDAC activity (*p* < 0.001) and the untreated cells show a slight increase in both HAT and HDAC activity, indicating that transfection might affect HDAC and HAT activity but not HDAC/HAT balance (no statistically significant difference). The *error bars* represent the standard deviation. Experiments were performed independently three times with n = 3 wells per treatment

In order to identify candidates for this shared regulation of the neuroprotective profile, we performed a further small RNA-seq experiment (see “Materials and methods”) to assay microRNAs (miRNAs) in our samples. We found that each diet group elicits its own specific miRNA profile (Fig. 3a), with switching from AL supplemented with LA to CR at 12 months leading to the greatest number of DE miRNAS (Table 4; for all DE miRNAs see Additional file 5). However, a single miRNA (miR-98-3p) was significantly overexpressed in all groups of CR rats and/or rats fed LA-supplemented diets (Fig. 3b). As mir-98 down-regulation in the cerebellum has been associated with AD and with neuroprotection [37, 38], we examined whether this miRNA could regulate the neuroprotective profile observed.

**Table 4:** Number of differentially expressed miRNAs in all diet comparisons

	Number of miRNAS DE
AL and CR ageing	
6 months AL versus 12 months AL	0
6 months AL versus 28 months AL	0
12 months AL versus 28 months AL	1
6 months CR versus 12 months CR	16
6 months CR versus 28 months CR	9
12 months CR versus 28 months CR	2
AL versus CR	
6 months AL versus 6 months CR	4
12 months AL versus 12 months CR	6
28 months AL versus 28 months CR	6
LA supplementation	
28 months AL versus 28 month AL + LA	3
28 months CR versus 28 months CR + LA	20
12 months AL versus 12 months AL + LA	5
Diet switching	
28 months AL versus 28 months AL > CR	4
28 months AL versus 28 months AL + LA > CR	39
28 months AL versus 28 months CR > AL + LA	26
28 months AL versus 28 months CR > AL	14
28 months CR versus 28 months AL + LA > CR	5
28 months CR versus 28 months AL > CR	7
28 months CR versus 28 months CR > AL + LA	2
28 months CR versus 28 months CR > AL	6

Benjamini-Hochberg FDR <0.05; n = 6 per group; 527 miRNAs expressed in total

To consider this question and relate it to our neuroprotective profile (Fig. 2a, c) induced by CR and LA diets, we investigated the role of miR-98-3p in HAT/HDAC homeostasis because changes in HAT/HDAC homeostasis have been noted as a potential cause of neurodegeneration [39]. Using CTX TNA2 cells (rat astrocytes derived from the cerebral cortex), we transfected miR-98-3p inhibitors and mimics, and measured HDAC and HAT activity (Fig. 3c). LA-supplemented cells showed a strong inhibition of HDAC activity as predicted from the structure of LA [40]. Increasing miR-98-3p (using a mimic) did not alter HDAC or HAT activity, suggesting that LA-induced inhibition of HDAC activity is not elicited through mir-98-3p. However, inhibition of miR-98-3p did up-regulate HDAC activity and greatly inhibited HAT activity. These results suggest that miR-98-3p is a candidate regulator of the neuroprotective profile by maintaining the HAT/HDAC balance and hence acetylation equilibrium.

### Defining the dietary memory effect of LA on longevity

**Fig. 4:**
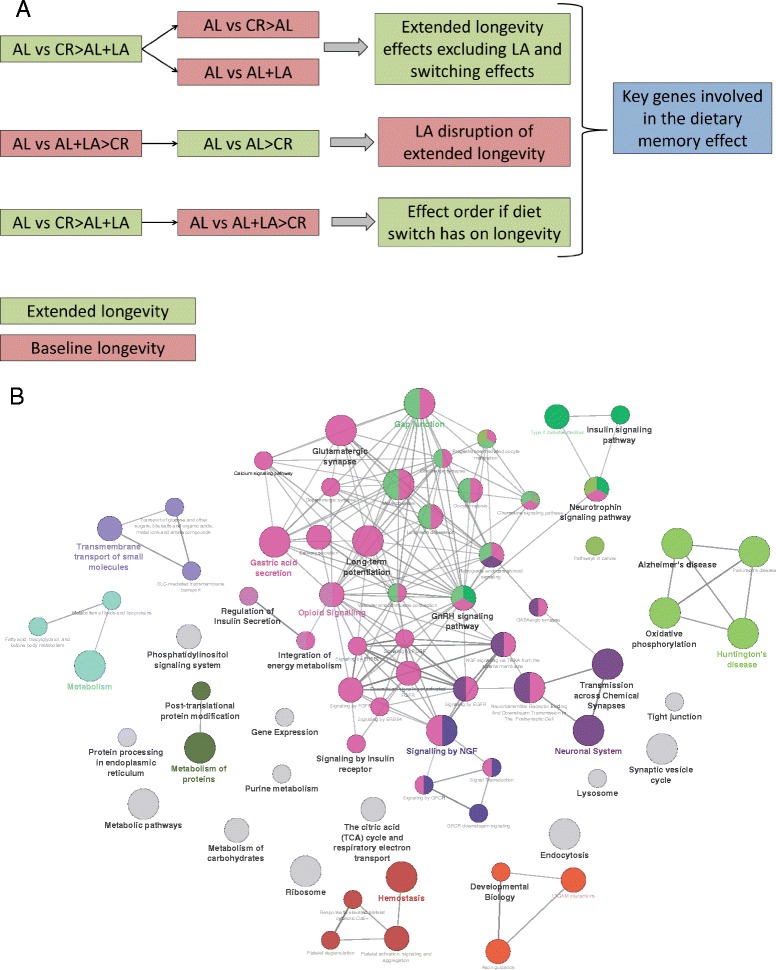
Dietary memory effect approach and networks. **a** The *red boxes* represent diet groups that have no effect on longevity (baseline longevity) and the *green boxes* represent diets that extend longevity. This shows how we compared the diet groups to identify the key genes involved in the dietary memory effect. First the DE gene list for AL versus CR > AL + LA (“>” = switched), which results in extended longevity, was compared with the DE gene lists for two conditions that do not result in extended longevity but do have a diet switch and LA supplementation, thereby identifying the genes for extended longevity but excluding the confounding effect of LA supplementation and diet switching. Next, DE genes from AL versus AL + LA > CR (baseline longevity) were compared with those from the extended longevity AL versus AL > CR condition, identifying genes LA induces that are important in disrupting the longevity effect of switching to CR. Finally, the effect of the order of LA supplementation (before or after diet switch) was identified. The three resulting lists were then compared to identify key genes involved in the dietary memory effect of LA. **b** Simplified networks of related statistically significant enriched GO terms using the Cytoscape add-on ClueGO [30, 31]. The network shows enrichment of gene function for the dietary memory effect genes (Additional file 6). The Cytoscape add-on ClueGO allows enrichment analysis and the collapsing of GO terms into parent categories for each comparison. The *filled coloured circles* (nodes) represent each statistically significant parent GO term. The *lines* (edges) between the nodes show that there are overlapping genes within each term. Each of the terms is statistically significant (Benjamini-Hochberg correction <0.05). The different sizes of the nodes relates to how many genes fall into that term

By far the most intriguing observation of Merry et al. [10] was the fact that LA cannot increase longevity alone but rats switching from CR to AL feeding supplemented with LA at 12 months of age retained the extended longevity characteristic of CR, despite being on an AL diet. Using our RNA-seq data we set out to disentangle the dietary memory effect of LA. Table 1 shows the mean survival of these rats on various feeding and dietary combinations and the number of DE genes compared with AL feeding. Figure 4a shows how we identified the extended longevity effect of CR switching to AL with LA. Briefly, we compared the lists of DE genes from the various cohorts in order to remove the confounding effects of diet switching and LA supplementation without extended longevity (Fig. 4a). This allowed us to focus on the genes associated specifically with the dietary memory effect (all DE genes are listed in Additional file 6). Dietary memory effect genes were enriched for the GO terms neurotrophin, insulin, phosphotidylinostol, NGF and GnRH signalling (Fig. 4b).

Genes involved in epigenetic regulation and specifically in the acetylation of histones are altered in the LA-supplemented long-lived groups. Examples include *Nur77* (*Nr4a1*), which regulates gene expression through acetylation [22], and *Morf4l1* (mortality like factor 1), which is involved in H2a and H4 acetylation and deacetylation (Additional file 6). These results suggest that histone acetylation is important in maintaining the CR longevity profile through LA supplementation. Further to this, other chromatin modifiers may play a role in the dietary memory effect (e.g. *Bag4*, *Mef2c Hdac5*, *Bcorl1*, *H3f3b*, *Brd3*, *Kat6a*, *Hist1h1d* and *Chmp1a*).

Genes involved in post-translational modification and metabolism of proteins are also enriched amongst genes potentially involved in the dietary memory effect (Fig. 4b). Examples include phosphatases/phosphorylases (*Pp1r16a*, *Pygb*, *Ppm1f*, *Camk1g*, *Dusp8*, *Dusp6* and *Dusp14* and a protein phosphatase inhibitor (*Ppi2*)) and kinases (*Pacsin1*, *Stk32c*, *Cdkn1a* (a.k.a. *p21*, a major player in cell senescence [41]), *Sik1*). This suggests that changes in the activity of target proteins through post-translational modification of non-histone proteins could be an important effect of LA supplementation and may play a role in its memory effect.

A previous study has noted a change in acetylation status of mitochondrial proteins with CR feeding [42]. Therefore, we also assessed non-histone protein acetylation using Western blotting (see “Materials and methods”). In our study we observed changes in global lysine acetylation status of proteins in the liver but not in the brain (Additional file 7). This suggests that post-translational modification of non-histone proteins may also be important, but possibly in a tissue- or protein-specific fashion.

### Diet-induced longevity effects: 137 genes are associated with long-lived cohorts

We next wanted to define the gene expression changes specific to increased longevity. By focusing on our dietary groups with extended longevity (Table 1) we were able to pinpoint “longevity genes” that are DE specifically in such groups. We also took into account the effect of LA supplementation regardless of the longevity effect, i.e., we excluded genes showing the same trend in expression in LA-fed groups with no increase in longevity. This gave us a “longevity gene” list of 137 genes (Additional file 8) which are changed when longevity is increased by our different diets. These genes are enriched for mitochondrion, oxidative phosphorylation, AD (ES = 2.57, not FDR significant) and neuron differentiation and development (ES = 1.99, not FDR significant). We also identified *Gad1* and *Grik2* as being longevity associated, linking glutamate excitotoxicity to ageing and longevity. Regeneration and repair related genes were also identified (*Swi5*, *Nrep* and *Smn1*) in long-lived cohorts.

**Fig. 5:**
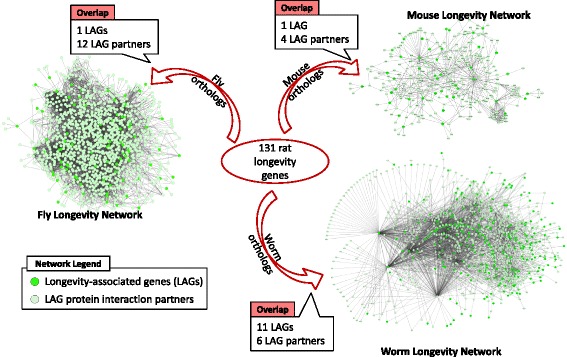
Overlap of longevity genes identified in the rat cerebral cortex with the longevity associated networks in mouse, *Drosophila* and *C. elegans.* The 28 longevity genes identified in rats in this study were found to be longevity-associated genes (*LAGs*) or partners of LAGs in the longevity networks for mouse, *Drosophila* and *C. elegans* (overlap significantly higher than expected by chance; *p* = 8.37E − 03, Fisher’s exact test). LAGs are genes which result in noticeable changes in the ageing phenotype and/or lifespan. These are identified experimentally through knockout, mutation, overexpression or RNA interference [76]. Longevity networks (previously described in detail [79]) are protein–protein interaction networks, include a core of LAGs, depicted in *green* in the figure, and their first order protein-interacting partners, shown in *light green*. The size of the network for each species is thus dependent on the protein–protein interaction data, but also on the number of LAGs available from the literature. All overlaps are higher than expected by chance (though this is not always statistically significant), with the overlap being significantly higher for worm LAGs, network partners of fly LAGs and network partners of mouse LAGs. Fisher’s exact test (one-tailed) *p* values for each of the overlaps are as follows: worm LAG *p* value = 6.11E − 05, fly LAG *p* value = 0.55, mouse LAG *p* value = 0.35, *p* value for LAG partners in worm longevity network = 0.09, *p* value for LAG partners in fly longevity network 0.027, *p* value for LAG partners in mouse longevity network = 0.034

To further investigate and validate our “longevity genes”, we mapped the orthologues of the rat genes to longevity-associated networks from mouse, *Drosophila* and *Caenorhabditis elegans* (see “Materials and methods”). Twenty-eight of our “longevity genes” were previously identified longevity-associated genes (LAGs) or partners of LAGs (Fig. 5; Additional file 9). The overlap with LAGs and their partners supports the idea that we have identified genes which have a role in longevity (overlap significantly higher than expected by chance; *p* = 8.37E − 03). Moreover, some of our “longevity genes” were found in multiple networks. For example, *Pik3r1*, which is involved in insulin resistance and AD [43], is found in the mouse, *Drosophila* and *C. elegans* networks, suggesting that it could be an important conserved modulator of longevity. *NPM1*, *Ubr5*, *Nduf6*, *Cox4i1* and *Cytochrome c oxidase subunit Va* are present in at least two of the networks, suggesting that these could also be important conserved modulators of longevity.

## Discussion

Our RNA-seq studies of the rat cerebral cortex during ageing and dietary manipulation of longevity provide insights at various levels. The cerebral cortex in ageing AL-fed rats is characterised by changes in synaptic density and vesicle transport that possibly affect synaptic transmission with increasing age. This fits with observations in humans of decreasing synaptic density after the age of 2–16 years, and a further decline between 74–90 years [44], and with models of neurodegeneration suggesting that age-related cognitive decline may be part of a continuum of neurodegenerative pathologies. We have found that key genes involved in DNA and histone methylation, transcription and chromatin re-modelling (e.g. the histone H3 methyltransferase *Dot1L*, *Tet3* and *Chd5*) are differentially expressed with age in rats. In agreement with published studies [45, 46], this suggests that ageing is characterised by a global reduction in methylation, possibly driving the gene expression changes observed. Importantly, these age-related changes in gene expression are not observed with ageing when the rats are maintained on a CR feeding regime.

We show that a shared neuroprotective gene expression profile is induced by a CR feeding regime and also by LA supplementation (when fed AL). This neuroprotective signature is characterised by an altered response to oxidative stress. Glutathione (GSH) is an important antioxidant, the metabolism of which is impaired in the brain during anxiety, in memory impairment states, and age-related neurodegeneration [34]. Glrx (a member of the antioxidant system and up-regulated in this study) is increased in AD brains and may be involved in beta-amyloid toxicity [47]. Transgenic mice that overexpress thioredoxin are more resistant to inflammation [48] and show decreased mortality rates in the early part of the lifespan but maximal lifespan is not increased [49]. As LA supplementation of the diet alone does not increase longevity, it is unlikely that thioredoxins will increase longevity. They are, however, likely to contribute to a neuroprotective phenotype and it is interesting that we found them to be overexpressed. This emphasises the potential importance of anti-oxidant and diet research in improving brain health in the elderly. We also observed gene expression changes in glutamatergic activity with age and diet. Changes in glutamatergic activity have been observed previously in the rat hypothalamus and hippocampus [50] and glutamate-dependent neuroglial calcium signalling has been shown to differ between the young and the adult brain [51]. To our knowledge, ours is the first study to indicate that CR feeding and LA dietary supplementation alter the glutamatergic response in the rat cerebral cortex. It is of note that LA can potentiate glutamate receptor activity and has been suggested as a neuroprotective therapy option for AD and related dementias [52–55].

In our work, we identified a number of differentially expressed miRNAs with age and dietary intervention. miRNAs are of interest because neuronal degeneration has been linked to miRNA dysfunction in *Drosophila* [56] and knock-out of DICER in the forebrain of adult mice results in a neurodegenerative phenotype [57]. miRNAs are also involved in synaptic development and plasticity in mammalian neurons [58] and have been implicated in neurological conditions such as Parkinson’s disease and AD [58]. One miRNA, miR-98-3p, showed consistent overexpression in CR and LA supplementation of the diet in our study. Using transfection and HAT and HDAC assays, we demonstrated a potential role for this miRNA in maintaining HDAC and HAT balance, thereby contributing to a neuroprotective phenotype. The activity of HDACs is altered in CR rats, suggesting that global deacetylation may be important in ageing, longevity and neuroprotection [59, 60]. Also, LA exhibits HDAC inhibitor activity even if this seems to be cell type-specific [61]. Our data support previous findings indicating that epigenetic modifications have a significant role to play in ageing and dietary modulation of ageing.

Using our unique set of RNA-seq data we began to unravel the dietary memory effect and gene expression changes related to increased longevity of LA supplementation of the diet. We saw an enrichment for genes related to the insulin signalling pathway, which was first associated with ageing and longevity in *C. elegans* [62] and is known to modulate ageing from worms to mammals [63]. Phosphatidylinositols are part of the insulin signalling pathway [64] and are also enriched in our results. Metabolism and energy-related pathways, such as the tricarboxylic acid cycle, are also enriched. It is interesting to note that the tricarboxylic acid cycle is known to mediate the epigenetic landscape of chromatin in cancer by regulating DNA and histone methylation and it has been hypothesised that epigenetic changes with ageing could be due to an impaired Kreb’s cycle [65].

Using existing data sets on LAGs, we found that 28 of our genes specifically DE in long-lived cohorts were known LAGs. It should be noted that LAGs are identified through single gene manipulations (knockout or overexpression), followed by lifespan studies. This shows that some of the genes that can modulate lifespan, and more importantly many of their protein–protein interaction partners, are differentially expressed in response to CR feeding.

## Conclusions

Additional studies are required to understand the role of epigenetics and post-translational modifications in ageing and longevity. This study presents extensive data on the effect of diet and ageing on the cerebral cortex transcriptome, serving as a starting point for future investigation into neuroprotection, longevity and dietary intervention in ageing and age-related disease. It also emphasises the importance of epigenetics and post-translational modification in ageing and longevity, and provides candidate genes for future studies.

## Materials and methods

### Diets

The non-supplemented CRM diet was supplied by Dietex International, Witham, UK. The LA-supplemented diet was the CRM diet enriched with a racemic mixture of DL-thioctic acid (α-lipoic acid) obtained from Fisher Scientific UK Ltd, at 1.5 g/kg by the Special Diet Systems Division of Dietex International. LA is known to have an anti-obesity effect by inhibiting food intake and retarding growth [66]; therefore, in the previous study by Merry et al. [10], it was ensured that the dose supplied to the animals did not inhibit the growth rate to induce an indirect dietary restriction effect.

### Animals

A previous experiment supplied the rat brain tissue for this study [10]. All animal husbandry procedures undertaken in this study were carried out in accordance with the provisions of the United Kingdom Animals (Scientific Procedures) Act 1986. The animal study from which the tissue was generated for the current publication was conducted under the Home Office Project Licence 40/2964, held by B.J.M. The work was also reviewed by the University of Liverpool Ethical Committee for Animal Welfare. Male BN rats (SubstrainBN/SsNOlaHSD) were obtained from Harlan UK at 21–28 days of age and maintained under barrier conditions on a 12-h light:12-h dark cycle (08:00–20:00). The health status of the rats was monitored at regular intervals through the screening of sentinel animals. All animals were caged in groups of four and fed the non-supplemented CRM diet AL until 2 months of age, when they were transferred to single housing and assigned randomly to one of six dietary groups as summarised in Table 1. All rats fed AL and CR were sacrificed at 6, 12, and 28 months of age. Animals subjected to CR were fed either the CRM diet or the LA-supplemented CRM diet at 55 % the daily food intake of control rats to maintain their body weight at approximately 55 % that of age matched AL-fed animals. The diet of animals maintained on a CR regime was supplied daily as pre-weighed rations between 10:30 and 11:00 h. The daily food ration supplied to CR animals was the same for age-matched animals irrespective of whether they were fed the CRM diet or the LA-supplemented CRM diet. Six dietary feeding combinations were studied to compare the effect of AL or CR feeding with and without LA supplementation. Survival trajectories for these groups were previously determined [10]. All groups were run simultaneously and so experienced identical husbandry and housing conditions. None of the animals exhibited any signs of pathology when sacrificed. Each age group had six rats from which tissue samples were taken, flash frozen and stored at −80 °C.

### Cortex dissection and RNA and miRNA extraction

To minimise thawing and therefore degradation of RNA, the cerebral cortex was removed from the whole brain on a solid CO_2_ base under a dissecting microscope. The cerebral cortex was cut into small pieces to aid RNA extraction.

RNA was extracted from the cerebral cortex using Qiagen's TissueLyser II and RNeasy lipid tissue kit. The quality of the extracted RNA was assessed using the Agilent 2100 Bioanalyser; all RNA integrity numbers (RINs) were above 8, indicating that good quality RNA had been extracted. Each sample was split in two for either whole transcriptome or small RNA analysis. For the whole transcriptome analysis the samples were pooled two by two (leaving three samples per age/diet group). Ribosomal RNA was removed from the pooled samples using a Eukaryote ribominus kit (Invitrogen) and confirmed with the Agilent 2100 Bioanalyser. Ribosomal removal, rather than poly(A) selection, allows certain non-coding RNAs without poly(A) tails to be included in the sequencing.

The miRNA fraction was enriched using the miRVana kit. The enriched miRNA samples were then pooled as with the whole transcriptome samples.

### cDNA library preparation and SOLiD sequencing

The whole transciptome library preparation protocol was carried out according to the manufacturer’s instructions. The RNA was fragmented and cleaned up using spin columns (Invitrogen) and SOLiD RNA adapters were then hybridised and ligated to the samples. Reverse transcription was performed to generate cDNA. The cDNA was purified, size selected, amplified and then purified again (as detailed in the SOLiD protocol). The size distribution of the cDNA library was assessed using the Bioanalyser. The samples were then subjected to emulsion PCR and sequenced in the Centre for Genomic Research at the University of Liverpool (https://www.liverpool.ac.uk/genomic-research/) using the SOLiD system 5500xl to generate 75-bp forward reads.

The miRNA library preparation protocol was carried out according to the manufacturer’s instructions. The samples were then subjected to emulsion PCR and sequenced in the Centre for Genomic Research at the University of Liverpool using the SOLiD system v4 to generate 50-bp forward reads.

### Whole transcriptome data mapping and differential expression analysis

The RNA-seq results from the SOLiD system were output as colour space fasta and quality files, and these files were mapped to the Ensembl release 71 rat reference genome (Rnor_5.0, March 2012) using Bowtie (http://bowtie-bio.sourceforge.net/index.shtml); the reads were filtered by quality using the –e phred quality setting (-e 400), multiple mapping reads were not allowed and the best option was used with mismatches limited to 2. For each sample approximately 36 million reads were generated. On average, 23 million reads per sample were mapped to the reference genome (approximately 63 % of reads generated were mapped; this figure is relatively low because of the conservative options specified in Bowtie but ensures that the highest quality and most robust alignments are reported). All data have been submitted to Gene Expression Omnibus (GEO) under the accession GSE57110. The differential expression analysis was carried out as in our previous study [14]. In brief, raw counts per gene were estimated by the Python script HTSeq count (http://www-huber.embl.de/users/anders/HTSeq/) and used by EdgeR [67] to estimate DE in pair-wise comparisons. Counts per million (cpm) were calculated and only genes with 1 cpm in at least three samples were included in the analysis. Trimmed mean of M-values (TMM) normalisation of the sequenced libraries was performed to remove effects due to differences in library size. EdgeR generates a FC for each gene; *p* values and the Benjamin-Hochberg FDR were calculated to statistically test the measured DE. As in previous studies, no effect of size cutoff was used, as ageing-related changes tend to be subtle [23].

### Enrichment analysis and heatmap generation

To create heatmaps of DE genes, R and the R package heatmap3 were used along with the log2 FC output from EdgeR. To assess the biological significance of gene expression changes we used the Cytoscape plug-in ClueGO to perform an enrichment analysis on each individual comparison and then, using a built-in algorithm, the GO terms were collapsed based on related terms and statistical significance in order to give a simplified network [68, 69]. Further enrichment analysis was performed by GSEA [70, 71] and DAVID [72].

### Whole transcriptome qPCR validation

To generate cDNA for qPCR, 3.5 μg of total RNA was reverse transcribed using Superscript III First-strand synthesis system for RT-PCR (Invitrogen, Paisley, UK). The Roche Universal Probe Library was used to design primers with sequences obtained from Ensembl. All primers were designed to cross an exon–exon boundary. The specificity of the primers was checked using BLAST (http://blast.ncbi.nlm.nih.gov/Blast.cgi). A reference gene experiment was conducted to identify the most stably expressed genes in the cerebral cortex with age and diet (data not shown); *Hprt1* (Additional file 10), *B2m* (Rn00560865_m1, Applied Biosystems) and *Ywhaz* (Rn00755072_m1 Applied Biosystems) were the most stably expressed in all conditions. The reference genes were used to normalise the qPCR results. Additional file 10 shows the primers, amplicons, and probes used. The qPCR assays were all performed in triplicate using a TaqMan™ ABI PRISM 7500 fast (Applied Biosystems, Foster City, CA, USA) in 96-well plate format. A 20-ml reaction volume was used per well, consisting of 10 μl Taqman 2× PCR master mix (Universal PCR Mastermix, Applied Biosystems), 0.2 μl each of 20 mM forward and reverse primers, 0.2 μl of 10 mM probe (Exiqon, Roche Diagnostics Ltd), 0.2 μl distilled water and 9.2 μl of cDNA or water for the negative controls. The amplification was performed as follows: 2 min at 50 °C, 10 min at 95 °C followed by 40 cycles of 95 °C for 15 s and 60 °C for 1 min. The efficiency of the assays was between 93 % and 107 % and the R2 values were >0.98. The ΔΔcT method was used to measure expression. The data were further corrected by the efficiency of the standard curve for each gene. Log2 FC was calculated and compared with the RNA-seq results in order to confirm the expression results. The standard error was calculated for log2 FC as follows: (Standard error/Mean) × log2e. For qPCR the relative quantification value was used to calculate standard error. For RNA-seq, raw reads converted into relative values were used to calculate standard errors.

### miRNA mapping

The RNA-seq results from the SOLiD system were output as colour space fasta and quality files. Using a custom script the base space adapter sequences was converted into colour space. A freely available Python script (cutadapt; https://cutadapt.readthedocs.org/en/stable/) was used to remove the adapter sequence and output a cfastq file. The freely available stand-alone Java program miRanalyzer [73] was used for mapping and analysis (http://bioinfo2.ugr.es/miRanalyzer/standalone.html). Using the Perl scripts provided by miRanalyzer, the reads were grouped and then mapped using Bowtie. miRanalyzer maps to a Bowtie index file for the rat; it then removes reads mapping to RefSeq (known mRNA) and Rfam (other known non-coding RNAs), leaving the user with reads that are likely to be miRNAs. New novel miRNAs are identified by miRanalyzer, detailing the secondary structure and number of times predicted by the five algorithms it uses. The results are also split into files for uniquely mapping and ambiguously mapping reads. This results in very clean results which are uniquely mapped.

### miRNA differential expression analysis

The differential expression analysis was carried out using a range of custom Python scripts, conditional quantile normalisation [74] and EdgeR. Conditional quantile normalisation and Lowess normalisation have been shown to be most appropriate for miRNA analysis [75]. The known miRNAs were analysed separately from the novel predictions. Only reads with one count per million in at least three samples were included in the analysis. After normalisation and exclusion of low read counts, the differential expression analysis was carried out using EdgeR’s generalised linear model and tag-wise dispersion. A FDR cutoff of 0.05 was used.

### miRNA qPCR

Validation of the miRNA differential expression results was carried out using the miScript PCR system (Qiagen). Reverse transcription using the miScript II RT kit was carried out according to the manufacturer’s instructions: 4 μl 5× miScript HiSpec buffer, 2 μl 10× miScript nucleics mix, 2 μl miScript reverse transcriptase mix into a total volume of 20 μl with 10 ng to 2 μg of template miRNA. This was incubated for 60 min at 37 °C and then 5 min at 95 °C. The resulting cDNA was diluted by adding 200 μl of distilled water. The miScript SYBR Green PCR Kit was used for the qPCR reaction. All samples were performed in triplicate in a 96-well plate format, using 2.5 μl cDNA, 12.5 μl 2× quantiTECT SYBR, 2.5 μl 10× miScript universal primers, 2.5 μl miScript primer assay, 5 μl RNase free water per well. Six small nucleolar RNAs (snoRNAs) provided and validated by Qiagen were used as the reference genes. Subsequently, a reference gene stability experiment ascertained that only three reference genes were required (*Snord96a*, *Snord95* and *Snord68*).

### LAG analysis

The comparison with known mouse, fly and worm LAGs was done using data from the Human Ageing Genomics Resources GenAge database, using build 16 [76]. Protein–protein interaction data for the construction of longevity networks and for the analysis of LAG partners were retrieved from the BioGRID database [77, 78], release 3.1.83. The construction of longevity networks has been described in detail previously [79]. Briefly, the networks include LAGs as a core set, and their first order interaction partners. Only the largest connected component is kept in the network.

In order to compare the rat “longevity genes” with genes from other species, orthologs were obtained using the InParanoid7 database [80]. Exclusion of inparalogs was done for the default threshold score of 0.05.

### Cell culture

CTX TNA2 cells were purchased from HPA cultures (UK). The cells were maintained in DMEM (Life Tech) with high glucose, L-glutamine, phenol red, sodium pyruvate. The DMEM was supplemented with 10 % foetal bovine serum and penicillin-streptomycin-glutamine. The cells were sub-cultured three times a week. Passages 12–14 were used for the experiments.

### Transfection

The confluent cells were sub-cultured and diluted 1 in 10 in antibiotic-free media and then counted using a haemocytometer. The cells were diluted to 1 × 10^4^ and 100 μl of cell solution was added to each well of a 96-well plate. The cells were incubated overnight at 37 °C, 5 % CO_2_ to allow them to attach. The transfection was performed using the Dharmacon miRNA system. Mimics and inhibitors for miR-98-3p, positive controls, including a miRNA mimic that inhibits GAPDH and a miRNA mimic that increases miR-16, and two negative controls (mimic and inhibitor) were used to assess the transfection efficiency and viability by comparing with a transfection reagent-only control and an untreated control. The viability was assessed using the Alamar Blue assay and by 96 hours the mimic was at 115 % viability compared with the transfection control, and the inhibitor was at 86 % viability compared with the transfection control (see Additional file 11 for results). The mRNA was extracted using the cells-to-CT kit (Life Tech) according to the manufacturer’s protocol. The miRNA was extracted using the miRvana kit as above. cDNA synthesis and qPCR were performed as above using *Actb* as the reference gene for the mRNA experiment. The transfection efficiency was ascertained by qPCR by measuring GAPDH and miR-16 knock down. GAPDH was reduced 4.2-fold and miR-16 was reduced 5.75-fold (see Additional file 11 for qPCR results).

### Nuclear extraction from cells and HAT and HDAC activity assay

The EpiQuik Nuclear Extraction Kit was used according to the manufacturer’s instructions to extract the nuclear proteins from transfected cells.

The Epigenase HDAC activity direct assay kit and the EpiQuik HAT activity kit (Cambridge Biosciences) were used to assess HAT and HDAC activity in transfected cells. The kit is an antibody-based assay with a colourmetric readout, assessed by the Multiskan microplate reader at 450 nm.

### Western blotting

Frozen liver tissue was defrosted and homogenised in an approximately equal volume of protein extraction buffer (50 mM Tris-HCl, pH 6.8, 86 nM 2-mercaptoethanol, 2 % sodium dodecyl sulfate (SDS) and general protease inhibitors (cocktail, Sigma, category no. P8340), centrifuged at 13,000 rpm for 15 min and the supernatant containing the soluble protein collected. The protein concentration of the supernatant was determined using a bicinchoninic acid assay according to the supplier's protocol (Abcam, Sigma) adapted to a 96-well plate format. Absorbance at 562 nm was measured and protein concentrations were back-calculated from the standard curve using the SpectroMax software (Molecular Dynamics). Samples were diluted to 5 mg protein ml^−1^.

SDS PAGE was carried out on a large BIO-RAD kit using a 7.5 % or 12 % SDS-polyacrylamide gel and containing 1.5 M Tris-HCl (pH 8.8). Two SDS-PAGE runs were carried out simultaneously in a standard 0.1 % SDS, Tris-Glycine running buffer. The mini gels were electrophoresed overnight at 45 V. Proteins were then transferred at 350 mA (4 hours) in transfer buffer (20 % methanol, Tris-Glycine buffer) onto a nitrocellulose membrane (Amersham Hybond ECL). Membranes were blocked with 5 % dried skimmed milk (Marvel) dissolved in Tris-buffered saline (TBS; pH 8.8). Membranes were then washed twice for 10 min in changes of TBS before being incubated for 1 hour with a 1/5000 dilution of mouse anti-acetylated lysine antibody (Ac-K-103, Cell Signalling Technologies). Membranes were incubated with the secondary horseradish peroxidise-conjugated anti-mouse antibody (Amersham ECL) at a 1/10,000 dilution in blocking solution (50 ml per membrane) for 1–2 hours. Visualisation of the protein bands was by enhanced chemiluminescence (ECL). The gels were stripped and re-probed with beta actin as a loading gel control.

The developed films were analysed using ChemiImager v4.4 software to give an arbitrary quantitative value to allow comparison of band intensity. This was adjusted to account for background, area and absolute protein concentration (by normalising to the loading gel control intensity).

### Data availability

All data have been submitted to GEO under the accession GSE57110.

### Ethics approval

The animal study from which the tissue was generated for the current publication was conducted under the Home Office Project Licence 40/2964, held by B.J.M. The work was also reviewed by the University of Liverpool Ethical Committee for Animal Welfare.

## Additional files

Additional file 1: Table S1. All the DAVID enrichments for each comparison. (XLSX 2288 kb) Additional file 2: Table S2. All genes DE with age. (XLSX 44 kb) Additional file 3: Figure S1. Enriched GO terms as a network generated from genes differentially expressed between AL and CR. (PDF 64 kb) Additional file 4: Table S3. The overlap between genes DE when feeding CR and LA dietary supplementation at 28 months of age when compared with AL feeding the non-supplemented diet. It also shows the DAVID enrichment of the overlaps. (XLSX 375 kb) Additional file 5: Table S4. All DE miRNAs for all diets and ages. (XLSX 17 kb) Additional file 6: Table S5. All genes altered in three comparisons. First, the extended longevity effect of CR switching to AL with lipoic acid. Next, the inhibitory effect lipoic acid had on extended longevity when switching from AL to CR feeding. Finally, the genes specific to CR feeding when switched to AL feeding with lipoic acid supplementation in comparison with the opposite switch which does not induce increased longevity. The DAVID enrichments are also included. (XLSX 157 kb) Additional file 7: Figure S2. Western blots for lysine acetylation in liver and in brain in multiple diet groups. (PDF 146 kb) Additional file 8: Table S6. The 137 longevity genes and the DAVID enrichments. (XLSX 48 kb) Additional file 9: Table S7. The 28 longevity genes that have been identified as longevity-associated genes (LAGs) or partners of LAGs. (XLSX 10 kb) Additional file 10: Table S8. The primers, amplicons, and probes used in the qPCRs. (XLSX 9 kb) Additional file 11: Data 1. The transfection efficiencies and viability after transfection with mimics and inhibitors. (XLSX 62 kb)

## Abbreviations

AD: Alzheimer's disease
AL: Ad libitum
CR: Caloric restriction
DE: Differentially expressed
FDR: False discovery rate
GEO: Gene Expression Omnibus
GO: Gene Ontology
GSEA: Gene set enrichment analysis
GST: Glutathione S-transferase
HAT: Histone acetyltransferase
HDAC: Histone deacetylase
LA: α-Lipoic acid
LAG: Longevity-associated gene
miRNA: MicroRNA
PCR: Polymerase chain reaction
qPCR: Quantitative PCR
SDS: sodium dodecyl sulfate
