# Supplementary figures and images for: Transcriptome analysis in calorie-restricted rats implicates epigenetic and post-translational mechanisms in neuroprotection and aging
Source: Genome Biol. 2015 Dec 22;16:285. doi: 10.1186/s13059-015-0847-2 (PMC4699360; doi:10.1186/s13059-015-0847-2)

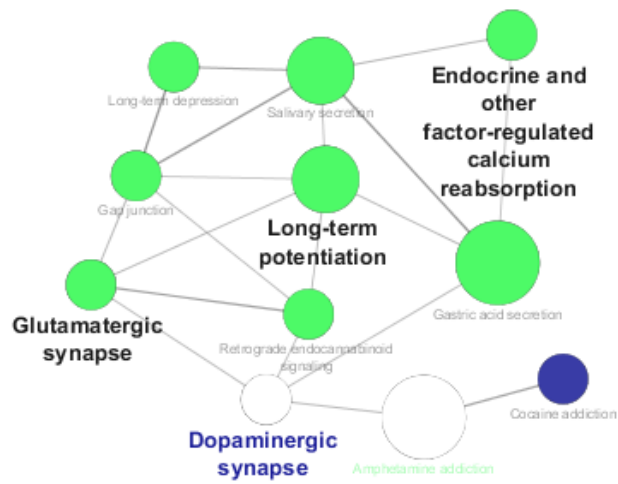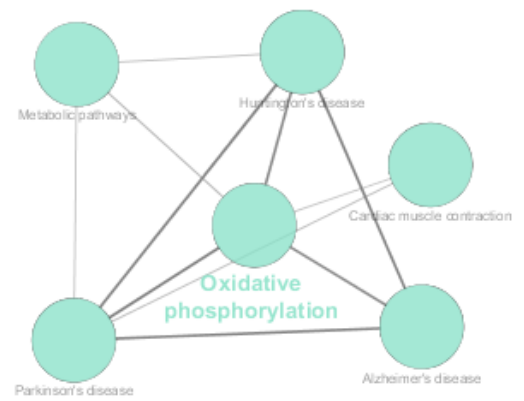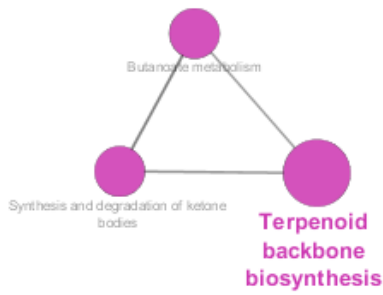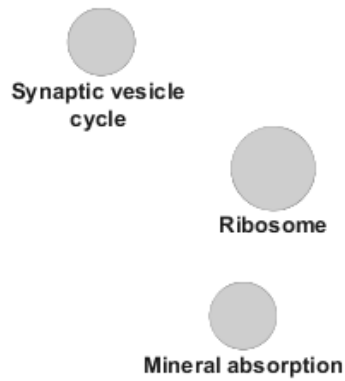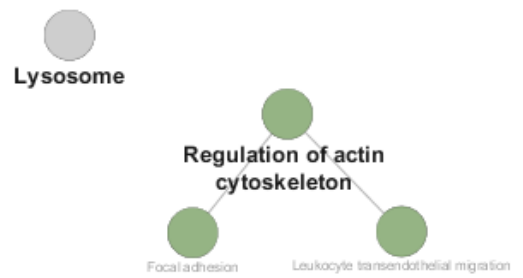

Supplement: Additional file 3: Figure S1. — Enriched GO terms as a network generated from genes differentially expressed between AL and CR. (PDF 64 kb) [file 13059_2015_847_MOESM3_ESM.pdf]

Liver

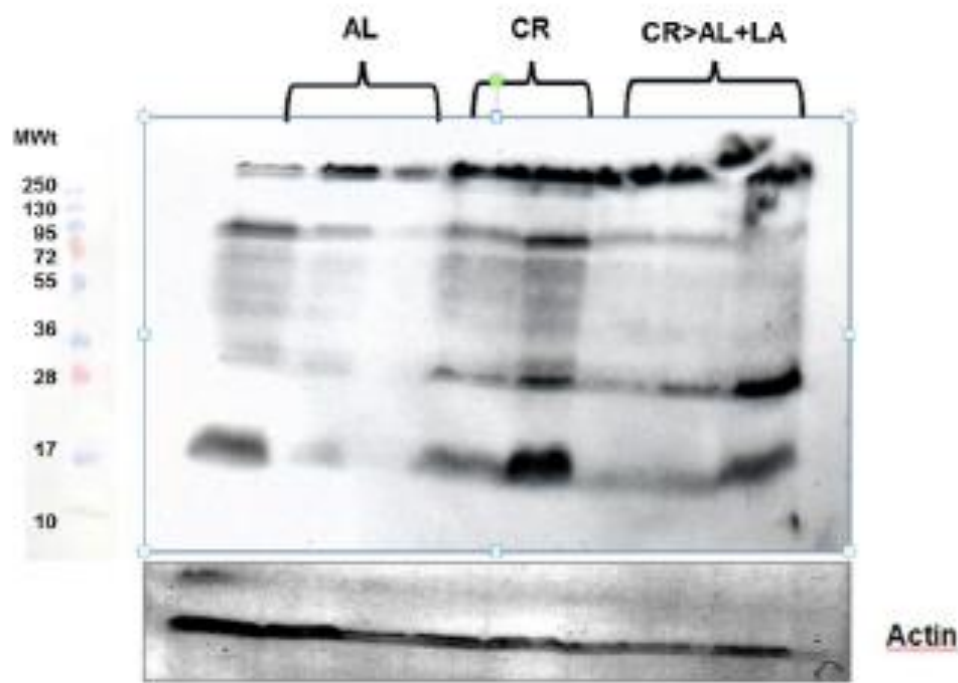

Brain – lysine acetylation blot

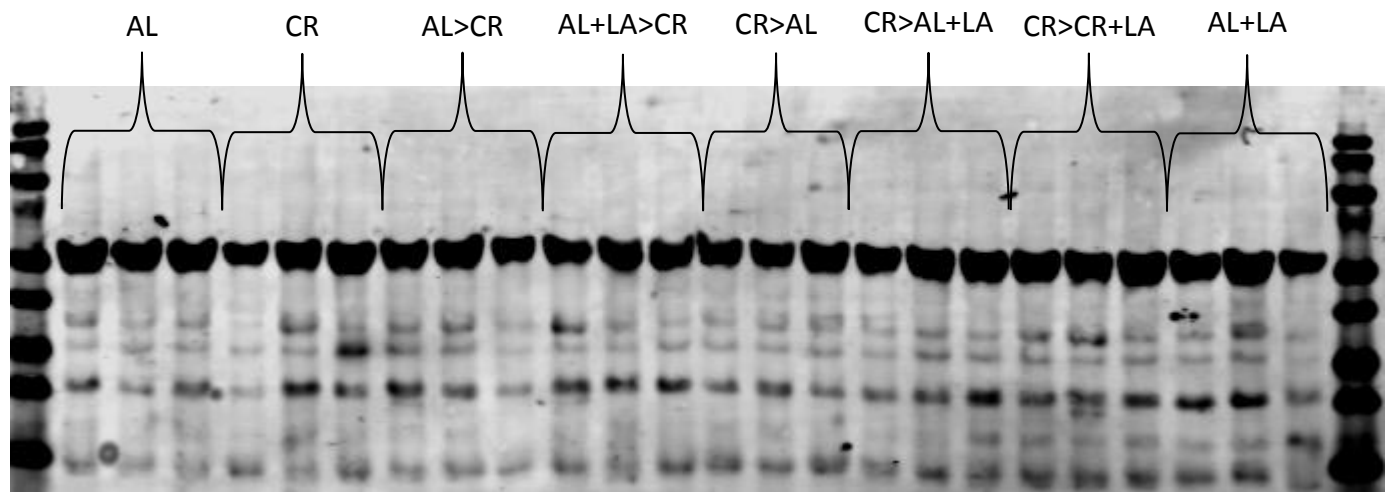

Brain – Coomassie gel for loading control

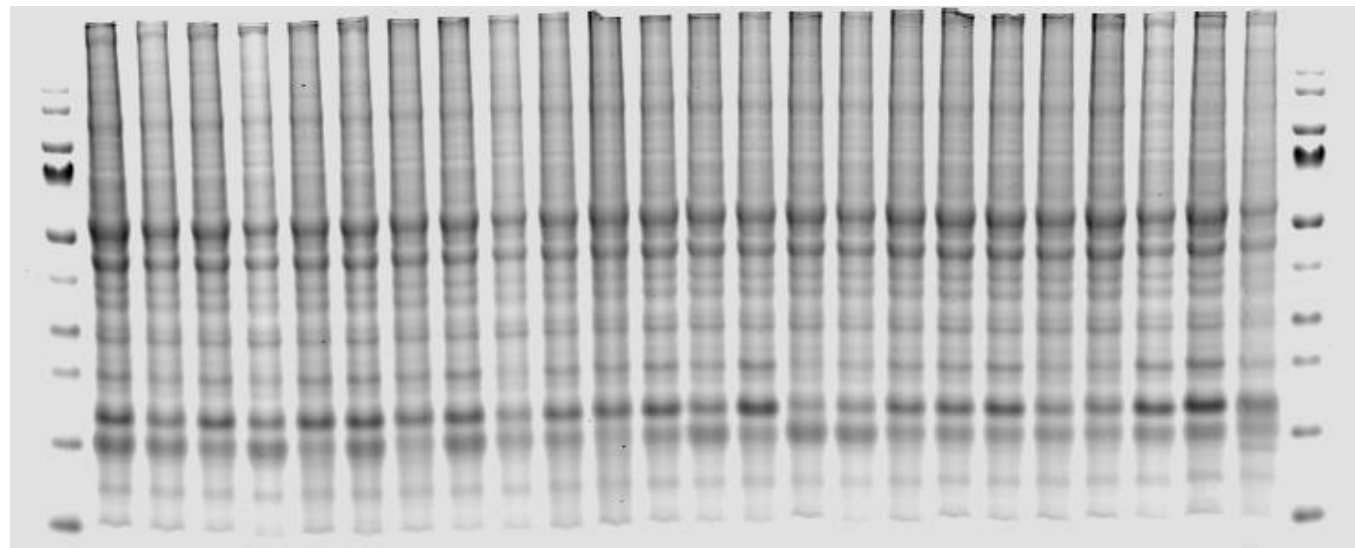

Supplement: Additional file 7: Figure S2. — Western blots for lysine acetylation in liver and in brain in multiple diet groups. (PDF 146 kb) [file 13059_2015_847_MOESM7_ESM.pdf]
